# Yeast-Based Vaccine Platforms: Applications and Key Insights from the COVID-19 Era

**DOI:** 10.3390/biom16010116

**Published:** 2026-01-08

**Authors:** Piyush Baindara, Roy Dinata, Ravinder Kumar

**Affiliations:** 1 Animal Science Research Center, Division of Animal Sciences, University of Missouri, Columbia, MO 65211, USA; 2 National Swine Testing Center, University of Missouri, Columbia, MO 65211, USA; 3 Department of Clinical Pharmacy & Translational Science, College of Pharmacy, University of Tennessee Health Science Center, Memphis, TN 38163, USA

**Keywords:** yeast-based vaccine, yeast surface display, SARS-CoV-2, COVID-19, pandemic preparedness

## Abstract

The COVID-19 pandemic accelerated vaccine innovation but also exposed weaknesses in global access and manufacturing. Yeast-based platforms, particularly *Saccharomyces cerevisiae* and *Pichia pastoris,* also known as *Komagataella phaffii*, offer a practical complement to vector systems. These eukaryotic microorganisms combine safety, scalability, and cost-effectiveness with the ability to express complex antigens and assemble virus-like particles. Building on the success of the recombinant hepatitis B vaccine, recent advances in glycoengineering, CRISPR-based host optimization, and surface display technologies have expanded the utility of yeast-based platforms for the rapid development of vaccines. Yeast-derived SARS-CoV-2 receptor-binding domain (RBD) subunit vaccines, such as Corbevax and Abdala (CIGB-66), demonstrate that affordable, immunogenic, and thermostable products are feasible at scale. Emerging innovations in glycan humanization, thermostable formulations, and oral or mucosal delivery highlight the potential of yeast-based vaccines for decentralized manufacturing and equitable pandemic preparedness. This review summarizes recent technical and clinical progress in yeast-based vaccine research, positioning these platforms as accessible and adaptable tools for future outbreak responses and global immunization strategies.

## 1. Introduction

The COVID-19 pandemic pressed the world into a race for vaccines. Within a year of the virus’s emergence, multiple vaccine platforms, including mRNA, adenovirus vectors, inactivated virus, and recombinant proteins, had entered deployment. That response was nothing short of historic, where the vaccination is estimated to have prevented more than 2.5 million deaths globally between the start of rollout in late 2020 and 2024, corresponding to approximately one death prevented per 5400 vaccine doses administered [1]. More recent modeling also suggests that over 2.5 million lives were saved during 2020–2024, even amid evolving variants and waning immunity [2,3]. Yet despite these achievements, the vaccine rollout revealed persistent weaknesses such as unequal access between high- and low-income countries, supply-chain bottlenecks, cold-chain logistics, and the challenge of updating formulations against variant strains. In that context, yeast-based vaccine platforms offer an intriguing alternative or complement. Yeasts, such as *S. cerevisiae* and *P. pastoris*, are well-established in the field and can express recombinant antigens or display immunogens on their surface. Over the past two decades, these systems have been explored for vaccines against viral infections, cancers, and other diseases [4,5]. More specifically, in the SARS-CoV-2 space, proof-of-concept studies have shown that a yeast-derived receptor-binding domain antigen can elicit broad immune responses in preclinical models [6]. Whole recombinant yeast vaccine designs (where yeast cells themselves carry antigen) are also being revisited for their simplicity and immunogenic potential [7]. Yeast-based vaccines, in particular, combine the advantages of safety, rapid scalability, and low manufacturing cost, positioning them as an important complement to cutting-edge genetic and viral platforms in building a more equitable and pandemic-ready vaccine ecosystem. Overall, yeast is one of the most underappreciated but powerful systems in modern vaccine development. Long known for its role in baking and brewing, yeast, especially *S. cerevisiae* and *P. pastoris*, has become a quiet workhorse in biotechnology. These organisms offer a rare combination of eukaryotic protein-processing ability, genetic simplicity, and scalability, making them ideal for producing safe and affordable vaccines [8]. Unlike mammalian cell systems, yeast can grow quickly on inexpensive media and at large scale, using existing fermentation infrastructure already available in many parts of the world. This makes yeast-based vaccines particularly appealing for global health settings where cost, cold-chain storage, and access to advanced biomanufacturing remain major barriers. Recent research has underscored yeast’s versatility. Yeast-expressed SARS-CoV-2 RBD vaccines have shown strong neutralizing antibody and T-cell responses in animal models, demonstrating immunogenicity comparable to more expensive expression systems [6,9]. Beyond subunit production, yeast can act as the vaccine itself, with whole recombinant yeast (WRY) systems presenting antigens directly on the yeast surface, sometimes triggering innate immune activation without added adjuvants [7]. Yeast surface display (YSD) and spore-based delivery are also gaining attention, offering stable, orally deliverable formulations that could bypass the need for needles and cold storage [10]. In short, yeast platforms marry biology with practicality as they are safe, well-characterized, and adaptable for rapid response to emerging pathogens. As the global vaccine landscape shifts toward greater decentralization and equity, yeast stands out as a platform capable of delivering both scientific rigor and public health reach, a low-cost, high-impact complement to next-generation vaccine technologies. In conclusion, by examining how yeast-based platforms contributed during COVID-19 efforts, this review aims to draw lessons for future pandemic vaccine preparedness. The hope is that yeast systems may help us build vaccines that are faster to manufacture, easier to distribute, and more equitable in reach.

## 2. Yeast as a Vaccine Production Platform

**Figure 1:**
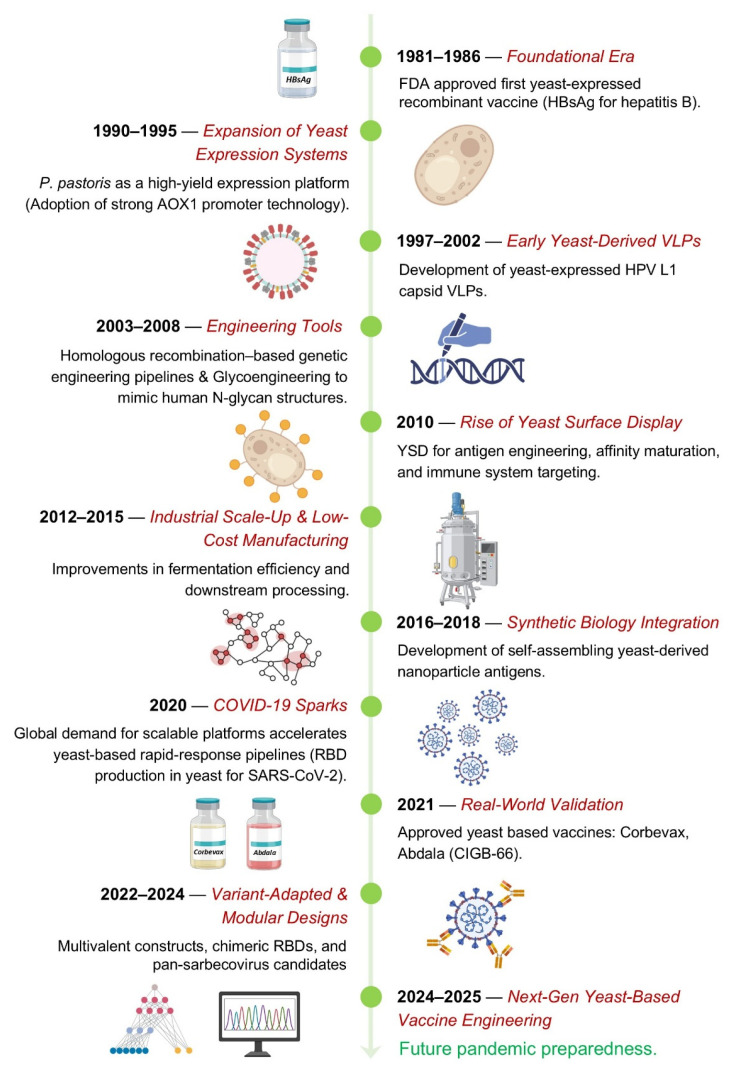
Milestones in the evolution of yeast-based vaccine platforms and their growing role in pandemic preparedness.

Yeasts such as *S. cerevisiae* and *P. pastoris* are attractive for vaccine development because they balance biological sophistication with industrial practicality. *S. cerevisiae* carries a long history of safe use. It is generally regarded as safe (GRAS), heavily studied, and accepted in food and pharmaceutical contexts, so its safety profile is well established [11,12]. Easy culture conditions, tolerance to stress such as high sugar, ethanol, osmotic changes, and robustness in industrial fermenters further strengthen its appeal. Further, *S. cerevisiae* has already been tested in contexts of antigen display and oral vaccines, such as expressing viral capsid proteins to induce systemic and mucosal immunity, with promising immunogenicity in animal models [13]. Additionally, *P. pastoris* offers complementary advantages. It grows to very high cell densities in relatively simple media, which translates into high yields per fermenter volume [14]. It also supports eukaryotic post-translational modifications, including glycosylation, disulfide bonds, and correct protein folding, critical for many antigens’ function and stability [15,16]. Because *P. pastoris* can secrete proteins into the culture medium, purification is simpler and often cheaper than intracellular expression systems [17]. More recently, engineered strains aim to humanize glycosylation, such as “GlycoSwitch” systems, to reduce undesirable yeast-type glycan patterns and improve compatibility with mammalian immunity [18]. Overall, when we combine a strong safety record, cost-efficiency, and scalability, these yeast species become powerful platforms (Table 1). They leverage existing fermentation infrastructure that many regions already have. Their flexibility helps shorten the path from gene to antigen, including fewer surprises in folding or expression, and their lower cost of goods helps make vaccine deployment more equitable. Taken together, today’s yeast platforms are not just cheap and scalable. They are increasingly precise, where one can choose promoters that fit facility constraints, route difficult antigens through a tuned secretory path, and sculpt N-/O-glycans to match immunological goals. This combination of industrial practicality with molecular control is exactly what vaccine development programs need when speed, cost, and equity matter altogether (Figure 1).

## 3. Hepatitis B Surface Antigen (HBsAg) Vaccine: A Proof of Concept

One of the strongest proofs that yeast can serve as a reliable vaccine production platform comes from the hepatitis B surface antigen (HBsAg) vaccine, the first recombinant human vaccine ever approved. Developed in the early 1980s, it replaced the older plasma-derived formulation with a recombinant version produced in *S. cerevisiae*. This breakthrough, commercialized as Recombivax-HB (Merck) and Engerix-B (GlaxoSmithKline), demonstrated that yeast could safely produce a complex viral antigen at an industrial scale without risk of blood-borne contamination [19]. The yeast-expressed HBsAg self-assembles into virus-like particles (VLPs), mimicking the structure of native virions and generating strong humoral responses in humans [20]. The success of the recombinant hepatitis B vaccine marked a turning point in vaccinology. It proved that eukaryotic microorganisms could produce safe, immunogenic, and scalable vaccines, laying the groundwork for today’s recombinant platforms. Decades later, yeast-derived HBsAg remains in global use, recommended by the World Health Organization as part of universal childhood immunization programs, with demonstrated long-term protection lasting more than 30 years [21]. The same principle has since been extended beyond hepatitis B, where *S. cerevisiae* has been engineered to express HIV-1, HPV, and tumor-associated antigens as whole recombinant yeast vaccines, capable of stimulating both antibody and cytotoxic T-cell responses through dendritic-cell activation [22,23]. These studies confirm yeast’s dual advantage that it can produce structurally authentic immunogens and act as its own adjuvant through innate immune receptor engagement. Together, these examples underscore yeast’s reliability, scalability, and adaptability. Overall, the legacy of HBsAg continues to validate yeast as a powerful, proven foundation for modern vaccine innovation from hepatitis B to next-generation platforms targeting SARS-CoV-2 and other emerging pathogens.

## 4. Yeast-Based SARS-CoV-2 Vaccine Approaches

### 4.1. Recombinant Protein Subunit Vaccines

**Figure 2:**
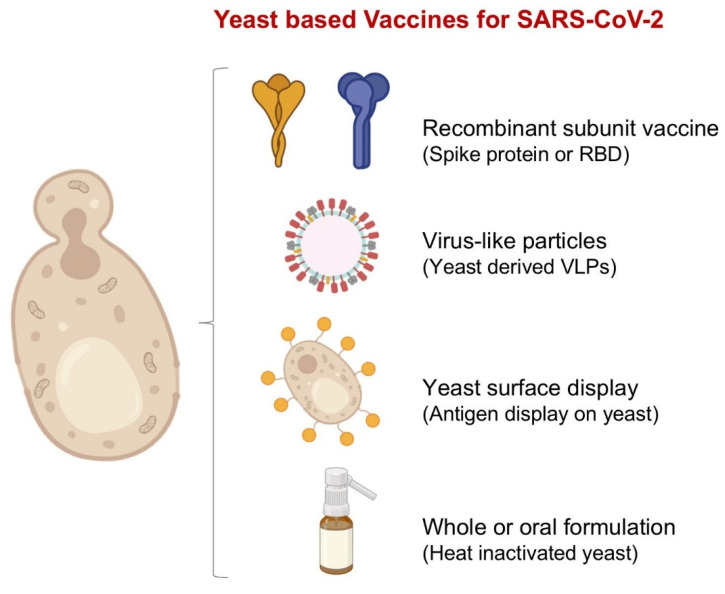
Yeast-based vaccine platforms developed for SARS-CoV-2. This figure highlights the major yeast-derived approaches used to generate SARS-CoV-2 vaccines. Yeast can produce recombinant subunit antigens such as Spike or RBD, assemble immunogenic VLPs, display viral epitopes directly on the cell surface, or be used as whole heat-inactivated preparations for oral or mucosal delivery.

One of the most straightforward ways to harness yeast for SARS-CoV-2 vaccines is by expressing the spike protein’s RBD or full ectodomain as a recombinant subunit (Figure 2). Yeast platforms can be engineered to produce large quantities of antigen, and then purify it and formulate it with adjuvants, a path that avoids some of the complexities of vector or nucleic acid systems. A clear proof-of-concept is the RBD219-N1C1 construct expressed in *P. pastoris*, which elicited both neutralizing antibodies and T-cell responses in mice [9]. In another study, a *P. pastoris*-derived RBD vaccine induced high binding antibody titers in mice and nonhuman primates, inhibited RBD-ACE2 binding, and showed neutralization of SARS-CoV-2 in vitro, while the antigen produced in yeast was structurally comparable to mammalian-cell versions despite glycosylation differences [24]. More recently, a yeast-expressed RBD vaccine formulated with Alum elicited cross-reactive neutralizing antibodies against multiple variants in mice, reinforcing the feasibility of yeast-based subunits for variant-adaptive immunization strategies [25]. Additionally, researchers have expressed RBD dimers in glycoengineered *P. pastoris* with reduced hypermannosylation to improve immune exposure and stability, while mice vaccinated with these dimeric antigens mounted strong antibody responses and protection in challenge models [26]. Also, YSD, a variant of subunit expression where RBD is anchored to the yeast cell wall, has been used for rapid screening and even as a potential oral immunogen, demonstrating mucosal and systemic responses in mice [27]. These studies showed how recombinant protein subunit strategies can bridge yeast’s advantages with real immunological impact. Overall, yeast offers scalable, lower-cost production, and by combining rational antigen engineering and glycoengineering, the expressed subunits can retain structural and antigenic integrity that provides a promising route for regional vaccine makers aiming for flexible, update-ready antigen platforms.

### 4.2. Virus-like Particles

Recombinant subunits are one path, and VLPs are the other big lever that yeasts give us. When a single viral protein self-assembles into a hollow shell, you get something that looks like a virus to the immune system but carries no genome. This assembly is important, as it provides repetitive, multivalent displays that drive strong B-cell activation, often with less antigen and simpler adjuvants (Figure 2). Yeast is a natural fit here because several capsid proteins spontaneously assemble when expressed in *S. cerevisiae* or *P. pastoris*, and fermentation scale-up is routine [28]. Yeast-made VLP precedents are already licensed and in the market. The HBsAg vaccine, first approved in 1986, consists of 22 nm HBsAg particles produced in *S. cerevisiae* and remains a global workhorse. Likewise, the first HPV vaccine (Gardasil) uses L1 capsid VLPs produced in yeast, validating that industrial yeast can deliver complex, immunogenic particles at scale. These successes are the historical proof that yeast VLPs are not just lab curiosities, but safe, manufacturable products [29]. For SARS-CoV-2, researchers have leaned on this foundation by using yeast-derived scaffolds as display platforms. One prominent approach decorates HBsAg VLPs with the coronavirus RBD using SpyTag/SpyCatcher chemistry, marrying the immunogenic geometry of the HBV particle (made in yeast) with a variant-updatable antigen insert. This hybrid design underscores a practical idea of keeping the scalable yeast VLP “chassis,” and swapping the antigen as the virus evolves [30]. Beyond coronaviruses, recent studies highlight how advanced yeast VLP manufacturing has progressed. Notably, *K. phaffii* has been used to produce VLPs for enteroviruses and poliovirus, including fully trivalent poliovirus VLPs made through controlled fermentation. These results show that high-density yeast cultures can reliably generate complex viral particles without ever working with infectious virus, a major advantage for safety and scalability. Overall, these pipelines are attractive for regional manufacturers because they cut biosafety risks and can leverage the existing fermentation capacity [31]. Taken altogether, yeast-derived VLPs combine the strengths that are needed in pandemic settings, including strong immunogenicity from ordered, repetitive antigen display, safer genome-free particles, and process economics that favor large, decentralized production.

### 4.3. Yeast Surface Display

YSD turns a cell into a tiny billboard where antigens are tethered to the yeast wall (classically via Aga1-Aga2) that can present hundreds of thousands of variants, available to stain with ligand or serum, and to sort the best by flow cytometry. This makes YSD a pillar for two jobs that matter in vaccine development (Figure 2). The first, is antigen design, including rapid screening to find spike or RBD constructs that fold well, bind with ACE2 as expected, and keep exposed epitopes neutralized. Studies using YSD to present SARS-CoV-2 RBD have shown how specific glycans and sites such as N331 and N343 shape ACE2 affinity and antibody recognition, giving direct, low-cost feedback for sequence and glycoform choices [32]. Second, immune profiling and discovery, which includes displaying wide antigen libraries against convalescent or vaccinee sera to identify potential escape mutations, as well as screening antibody or mini-binder libraries to isolate high-affinity, variant-resilient binders [33]. Further, recent engineering upgrades have made YSD more modular and production-friendly. SpyTag/SpyCatcher chemistry allows for “plug-and-display” purified antigens onto the yeast surface without re-cloning, useful for quickly swapping variant RBDs or multimerizing domains, while improved Spy systems and ligation controls have tightened this workflow [34]. Beyond bench screening, when antigens are displayed in *P. pastoris* or *S. cerevisiae* and given orally, they can trigger systemic and mucosal responses in mice, pointing to needle-free, cold-chain-light options that fit equity goals. Several recent studies report IgG and fecal IgA responses and reassuring gut histology after oral dosing of yeast displaying multi-epitope SARS-CoV-2 constructs or spore-based displays [35].

Taking all these findings together, YSD gives vaccine researchers a fast loop from idea to insight, optimizes the antigen, visualizes what serum “sees,” and even prototypes low-cost oral candidates on the same platform where speed, scalability, and access all matter, along with a blend of screening power and translational promise.

### 4.4. Oral Yeast-Based Vaccines

Oral whole-yeast vaccines take a simple idea and make it practical by expressing the antigen inside *S. cerevisiae* or *K. phaffii*, heat-inactivating the cells, and letting the yeast’s own wall act as the delivery vehicle and adjuvant (Figure 2). Yeast β-glucans target pattern-recognition receptors like Dectin-1, helping gut-associated immune tissues to handle the payload and mount mucosal and systemic responses without handling live vectors or replicating pathogens [36]. In preclinical models, oral dosing with recombinant, heat-inactivated yeast has generated robust IgG and IgA responses and, in some constructs, neutralizing activity, including recent work using *Pichia* that displays multi-epitope SARS-CoV-2 antigens for oral delivery [35]. Yeast spore-based formats push stability even further, where spores tolerate harsh gastrointestinal conditions and can present antigens on their surface, pointing to cold-chain-light vaccines that are easy to store and ship [10].

This approach is also supported by clinical experience with whole recombinant, heat-inactivated *S. cerevisiae* in oncology, such as GI-4000, which showed acceptable safety and measurable antigen-specific T-cell responses in humans, evidence that the platform can be manufactured consistently and stimulate meaningful immunity in people [37]. Conceptually, oral whole-yeast vaccines line up well with equity goals as they are inexpensive to make at scale, needle-free, and compatible with decentralized production. Overall, it is clear that better antigen design, glyco- and surface-engineering, and optimized inactivation or encapsulation are turning yeast from a delivery curiosity into a realistic oral vaccine platform.

## 5. CRISPR-Based Engineering of Yeast Strains for Vaccine Antigen Design

CRISPR has turned *S. cerevisiae* and *P. pastoris* into faster, more precise chassis for vaccine antigens. In *P. pastoris*, streamlined Cas9 toolkits now enable scarless knock-ins, marker recycling, and high editing efficiency using short ssDNA donors. These capabilities are especially useful when fine-tuning Spike or RBD constructs for better yield and stability [38,39]. CRISPR has also been pushed beyond “one-off” edits, such as rDNA landing pads, which enable multi-copy integration for high-titer expression, while inducible editing schemes boost homologous recombination results into clean, multi-gene constructs [40]. Next, base editors were introduced into *P. pastoris* to perform efficient C-to-T conversions without creating double-strand breaks. This makes it easy to adjust N-linked glycosylation sites or fine-tune signal peptides and protease-cleavage motifs that influence secretion [41]. Prime editing, recently improved by directed evolution in yeast, adds precise small insertions or deletions and point changes, letting teams prototype stabilizing substitutions or epitope-exposing tweaks without donor templates [42]. Together with the glycoengineering, these tools co-optimize sequence, folding, secretion, and glycan presentation in the same host.

Transcriptional control has also advanced quickly. In *S. cerevisiae*, CRISPRi/a libraries make it easy to tune the expression of chaperones, trafficking proteins, or lipid-metabolism genes and provide the opportunity to choose the best supporting combinations for a given antigen. New dual dCas9/dCpf1 CRISPRa/i systems add another layer, giving researchers independent options to adjust the expression of multiple genes at once [43]. In *P. pastoris*, genome-wide CRISPR screening is emerging, opening the door to unbiased host-factor discovery for difficult antigens [44]. Further, open, license-light nuclease options like MAD7 help on the equity side by reducing IP friction for regional manufacturers, and community resources such as OPENPichia provide a well-characterized chassis and modular expression toolkit that pair well with CRISPR workflows [45,46]. In short, researchers can now drop variant-specific antigen cassettes into safe-harbor sites at multi-copy, humanize glycans, boost secretion, and test stabilizing mutations within days. CRISPRi/a tools then make it easy to see which host edits raise titers or improve antigen quality, while the same yeast strains can be used to map serum binding and escape profiles. Overall, this is a tight design–build–test loop that serves COVID-19 today and general pandemic preparedness tomorrow.

## 6. Yeast-Derived Adjuvants and Immunostimulatory β-Glucans

Yeast cell walls are built around β-1,3/1,6-glucans that our innate immune system recognizes through receptors like Dectin-1, complement receptor 3, and TLR2/6. When formulated as purified particles or carried within heat-inactivated yeast, these glycans act as natural danger signals that recruit and train phagocytes, amplify antigen presentation, and shape downstream B- and T-cell responses. This has been demonstrated in recent work, including Dectin-1-dependent activation and cytokine programs, where it gives yeast materials a clear mechanistic footing as vaccine adjuvants [47]. On the practical side, glucan particles (GPs) from *S. cerevisiae* have matured into a modular platform, hollow β-glucan shells that can encapsulate protein or nucleic-acid antigens and simultaneously provide adjuvant activity. Next, biocompatibility, macrophage and dendritic-cell targeting, and dose-sparing effects in multiple pathogen models, including formulation studies, map how particle size and composition tune immune readouts has been reviewed recently [48]. Further, β-Glucans also engage in trained immunity, where short exposures reprogram monocytes and other innate cells through metabolic and epigenetic changes, boosting responses to later challenges. Additionally, heat-killed *S. cerevisiae* behaves as a Dectin-1 agonist and has shown adjuvant-like effects on B-cell class switching in preclinical systems. Clinically, soluble yeast β-glucan has reached oncology trials, where it reproducibly primes innate cells and pairs safely with monoclonal antibodies. These studies do not replace vaccine trials, but they underscore the manufacturability and immunologic activity of clinical-grade yeast β-glucans [36]. Overall, yeast-derived β-glucans give protein subunit and VLP vaccines a native adjuvant option that is mechanistically clean, scalable, and compatible with decentralized production. As we refine the connection between glycan structure, particle engineering, and trained-immunity signatures, they look increasingly relevant for COVID-19 boosters and for the next fast-moving respiratory threat [49].

## 7. Stability and Thermostability Advantages

Cold chains are fragile, expensive, and easy to break. In many low- and middle-income settings, this translates into missed vaccinations and wasted doses. WHO’s “controlled temperature chain” (CTC) framework was built to ease that burden by allowing certain vaccines to be used safely above 2–8 °C for defined periods. Yeast platforms fit this reality. Heat-inactivated or lyophilized whole-yeast formulations protect antigens inside a sturdy cell wall of β-glucans and mannans, which acts like a built-in stabilizer. In a proof-of-concept study, proteins retained integrity for a year or more when stored in freeze-dried *Saccharomyces* at 30–37 °C, a temperature window that maps to last-mile conditions in many regions [50]. Yeast spores display further durability, where spores tolerate heat, desiccation, and low pH, and can present antigens on their surface, useful for oral or ambient-stable formats being explored for enteric and respiratory pathogens [10]. Recombinant particles are also beneficial, as they are naturally robust, and they can be further strengthened by engineered disulfides and by lyophilization or spray-drying, with multiple studies showing months of stability at ambient and even elevated temperatures. These features make VLP-based vaccines good candidates for CTC use and for decentralized stockpiles [51]. The bottom line is practical, if a vaccine can tolerate time out of the fridge, outreach becomes easier. Health workers can carry doses farther, weekend clinics are less risky, and local manufacturers can move product without deep-freeze shipping. Overall, thermostability improves reliability and lowers cost at the same time, which is exactly what programs in LMICs need.

## 8. Comparison with Other Platforms

### 8.1. Yeast vs. mRNA, Adenoviral Vectors, Mammalian Cell Culture, and Insect-Cell VLPs

Each platform solves a different part of the pandemic-preparedness puzzle. Yeast (*S. cerevisiae* and *P. pastoris*) is attractive when you need low cost, straightforward scale-up, and cold-chain requirements. Recombinant antigens and VLPs can be brewed in stainless-steel fermenters that many countries already run for biologics and enzymes, and then purified with familiar downstream steps. The track record is real: today’s hepatitis B vaccines express HBsAg in yeast, and newer products even use *Hansenula polymorpha* or *S. cerevisiae*, which keeps regulatory risk modest and costs predictable (FDA/CDC labels). Yeast walls also carry innate immunostimulants, and the field is moving on glycoengineering to reduce hypermannose and approach human-like glycans. In practical terms, this makes yeast well-suited for affordable boosters, thermostable formats, and regional manufacturing where capital is tight [4].

On the other hand, mRNA wins on speed and agility. Once a sequence drops, the design to clinical material can be measured in weeks, and production is cell-free, which simplifies tech transfer. Important tradeoffs are storage and formulation. Lipid-nanoparticle products still require frozen storage before thaw, though recent labels allow a longer time at 2–8 °C after thaw, which eases logistics without eliminating them. In short, mRNA is ideal for the first wave and rapid updates, while the cold chain and input costs can strain equity unless supply chains are primed [52]. Adenoviral vectors remain a strong option because they can drive durable T-cell immunity, work with simple dosing, and many formulations stay stable at standard refrigerator temperatures. They are also straightforward to manufacture at scale in cell culture, which makes global distribution more practical. Still, two issues shape how they should be used in preparedness plans. First, pre-existing immunity to the vector can reduce vaccine uptake in some populations. Second, a very small number of thrombosis-with-thrombocytopenia cases reported during COVID-19 affected the perceived risk–benefit balance for this platform. Along with these clinical considerations, adenoviral production requires more specialized facilities and higher biosafety standards than microbial platforms, which adds cost. Even so, as part of a layered response, especially after a rapid mRNA rollout, they continue to be an important and reliable tool, provided programs plan for these constraints [53]. Further, mammalian cell culture (especially CHO cells) is the major driver when you need human-like post-translational modifications and complex glycoproteins. That fidelity matters for some antigens and can improve functional immunogenicity. The downside of this is cost and time, where building and running GMP CHO capacity is expensive, and scale-up timelines are longer than microbial systems. Nevertheless, the regulatory track record is excellent, and several modern vaccines use CHO-derived antigens. Overall, in a diversified strategy, mammalian cells anchor high-complexity antigens and combination vaccines once programs move beyond the emergency phase [54]. Insect-cell platforms with baculovirus (BEVS) are proven helpful for both subunits and VLPs. They scale quickly, have broad regulatory acceptance, and deliver refrigerator-stable products. NVX-CoV2373 and Flublok are concrete examples with strong clinical data along with 2–8 °C storage, which is a plus for global distribution. One scientific drawback is glycosylation, where insect cells tend to make paucimannose N-glycans rather than the complex, sialylated forms seen in humans. However, it does not prevent efficacy, but it can influence antigenicity and requires thoughtful design. Overall, insect cells are a solid middle ground on speed, manufacturability, and cold-chain fit for vaccine development [55]. Putting together, diversity creates resilience. mRNA gives you the earliest shots in arms, adenoviral vectors provide potent cellular immunity with simple storage, and mammalian cells serve complex, glycan-sensitive antigens, insect cells deliver fast, fridge-stable protein vaccines, while yeast brings affordability, scalable stainless-steel capacity, and an easier path to thermostable or controlled-temperature-chain use. Overall, for equitable access, anything that avoids ultra-cold storage and leverages existing bioreactors in low- and middle-income countries is a win. Especially, yeast and insect-cell programs pair naturally with CTC strategies, while mRNA and adenoviral programs benefit from regional fill-finish and clear communication about storage (Table 2). A portfolio strategy that mixes these platforms will shorten the time from sequence to dose, lower cost per dose, and widen geographic manufacturing footprints.

### 8.2. Manufacturing Speed, Cost, Cold-Chain Requirements, and Safety Profiles

Manufacturing speed, production cost, cold-chain requirements, and other safety issues are major factors in vaccine development. If one talks about the speed, mRNA is still the quickest to stand up. During COVID-19, the first US-based Phase I trial began 66 days, just after the first SARS-CoV-2 genome sequence was posted, illustrating how fast a sequence can become clinical material when the platform and supply chain are ready [56]. Adenoviral vectors and protein subunits, including insect-cell VLPs and yeast-made antigens, move a little slower because they rely on cell-based expression and purification campaigns, though “platformized” processes have shortened these timelines. Novavax’s protein vaccine is a good example of how a mature subunit process can scale quickly once the antigen design is locked [57]. On the other hand, comparative techno-economic work suggests that material and capital costs tend to be higher for mRNA vaccines than for traditional protein subunits and VLPs, largely because of specialized raw materials and formulation steps. Notably, microbial platforms like yeast benefited from inexpensive media and high cell densities, which helps with the cost of goods for protein antigens [58]. Finally, storage often drives last-mile feasibility. Especially, mRNA products have improved but still rely on frozen storage before use, with limited time permitted at 2–8 °C after thaw [59]. On the other hand, protein subunit vaccines such as Novavax’s NVX-CoV2373 can be stored at 2–8 °C with a 9-month shelf life, which is a clear advantage for outreach [60]. Next, inactivated and recombinant influenza vaccines made in insect or mammalian cells, such as Flublok, could also be stored at 2–8 °C for a longer time period [61]. Interestingly, yeast is an attractive alternative here as whole-yeast and spore-display formats can be lyophilized and remain stable for months at room temperature or even 30–37 °C in proof-of-concept studies, aligning with WHO’s CTC principles used for field campaigns [50]. Next, all platforms have strong benefit-risk profiles when used appropriately. mRNA vaccines have a known but rare risk of myocarditis, highest in young males, which public health agencies monitor closely [62]. Adenoviral vectors carry a rare risk of vaccine-induced immune thrombotic thrombocytopenia (VITT), which is now well characterized in the literature and labeling [63]. Protein subunits have a long safety track record. NVX-CoV2373 is refrigerator-stable with a generally reassuring safety profile, though rare myocarditis cases have been reported [64]. Yeast-derived antigens are also well established clinically through decades of hepatitis B vaccine use, with safety details reflected in FDA package inserts (Recombivax-HB and Engerix-B) and subsequent reviews [57]. Overall, if the priority is getting first doses out quickly, mRNA is still the fastest option. Next, for broad distribution in low-resource settings, vaccines that tolerate refrigeration or ambient temperatures are a much better fit. Protein vaccines made in yeast or insect cells already meet these needs, and yeast’s potential for true thermostability and ultra-low production costs makes it especially attractive for regional manufacturing and emergency stockpiles (Table 2). Conclusively, a diversified portfolio can let programs start fast and then shift to scalable, affordable, easier-to-ship vaccines as supply grows.

## 9. Where Does Yeast Stand Out?

Yeast is popular with process engineers for a simple reason: it keeps costs down. Comparative techno-economic work finds that recombinant DNA platforms using microbial hosts tend to hit lower minimum selling prices than more capital-intensive cell-culture systems, largely because media, facilities, and raw materials are cheaper and runs are shorter [58]. Yeast has been suggested as a cost-effective way to make vaccine antigens of quality, especially when mammalian glycosylation is not essential [65]. Practically, *P. pastoris* and *S. cerevisiae* grow to high cell density on simple media, which lifts volumetric productivity and helps reduce the cost of goods. Moreover, whole-yeast formats can be heat-inactivated and given orally, with the yeast wall’s β-glucans acting as built-in adjuvant signals to the gut immune system. Recent studies using *P. pastoris* engineered to display SARS-CoV-2 epitopes showed that oral administration in mice induced both systemic IgG and mucosal IgA responses, including fecal IgA, without causing gastrointestinal pathology [35]. Yeast spores push the idea further as they tolerate heat, acid, and desiccation, and can present antigens on their surface, which fits last-mile realities where refrigeration is scarce [10]. Yeast production also fits easily into existing manufacturing infrastructure, as it uses the same stainless-steel fermenters already deployed worldwide for enzymes and other biologics. Moreover, *P. pastoris* supports high-density fermentation and efficient secretion, making scale-up relatively straightforward even for complex vaccine formats. This capability has been demonstrated for poliovirus VLPs, including fully trivalent particles produced reliably through controlled yeast fermentation [66]. Recent studies emphasize that microbial systems are among the fastest to bring online when new capacity is needed, which matters in an outbreak [67]. Additionally, coupled with the WHO CTC framework, yeast-based products that tolerate ambient conditions can further ease distribution in low- and middle-income countries. Overall, yeast-based vaccines are the most suitable options for vaccine development with affordable doses, a realistic path to oral or room-temperature formats, and manufacturing that can be stood up or localized quickly.

### Case Studies and Recent Progress

Yeast-based SARS-CoV-2 vaccines have now moved far beyond proof-of-concept, with multiple candidates demonstrating clinical viability, large-scale manufacturability, and real-world impact. Corbevax is a yeast-based protein subunit vaccine (*Pichia*-expressed RBDs), developed by Texas Children’s Hospital Center for Vaccine Development and Baylor College of Medicine in Houston, Texas, and Dynavax Technologies based in Emeryville, California. Notably, Corbevax completed Phase I/II and II/III trials with strong neutralizing responses and favorable safety, ultimately earning the WHO emergency use listing in 2024 [68,69]. Later advancing variant-updated XBB.1.5 booster studies, making Corbevax a vibrant example of how a low-cost, stainless-steel-fermented yeast-based vaccine can scale globally. Next, Cuba’s Abdala, another *Pichia*-expressed RBD vaccine, delivered 92.3% efficacy in a 48,290-participant Phase III clinical trial and maintained high real-world effectiveness against severe outcomes during the Delta wave [70]. This underscores that yeast can meet both efficacy and operational demands with simple 2–8 °C storage. Further, Abdala’s companion intranasal candidate, Mambisa, showcased the versatility of the yeast-based vaccine platform by safely boosting antibody responses in convalescent plasma in Phase I and II testing [71]. Additionally, preclinical yeast RBD programs, including RBD219-N1C1 and RBD203-N1, laid the groundwork for these successes by demonstrating manufacturability, potent neutralization, and robust Th1-biased responses [72]. Adding to this, more recent studies have expanded to RBD monomers, genetically linked dimers, and XBB.1.5-targeted constructs, including versions produced in glycoengineered *Pichia* strains [6,73]. Moreover, early efforts in oral or surface-display yeast formats also point toward needle-free, cold-chain-light strategies aligned with global access [35]. Taken altogether, clinical and preclinical results demonstrate that yeast is no longer an experimental vaccine platform, while it is a flexible, scalable, variant-ready system that can support both national immunization programs and equitable global preparedness (Table 3).

## 10. Future Directions and Conclusions

### 10.1. Potential of Yeast-Based Vaccines for Pan-Coronavirus or Variant-Proof Designs

**Figure 3:**
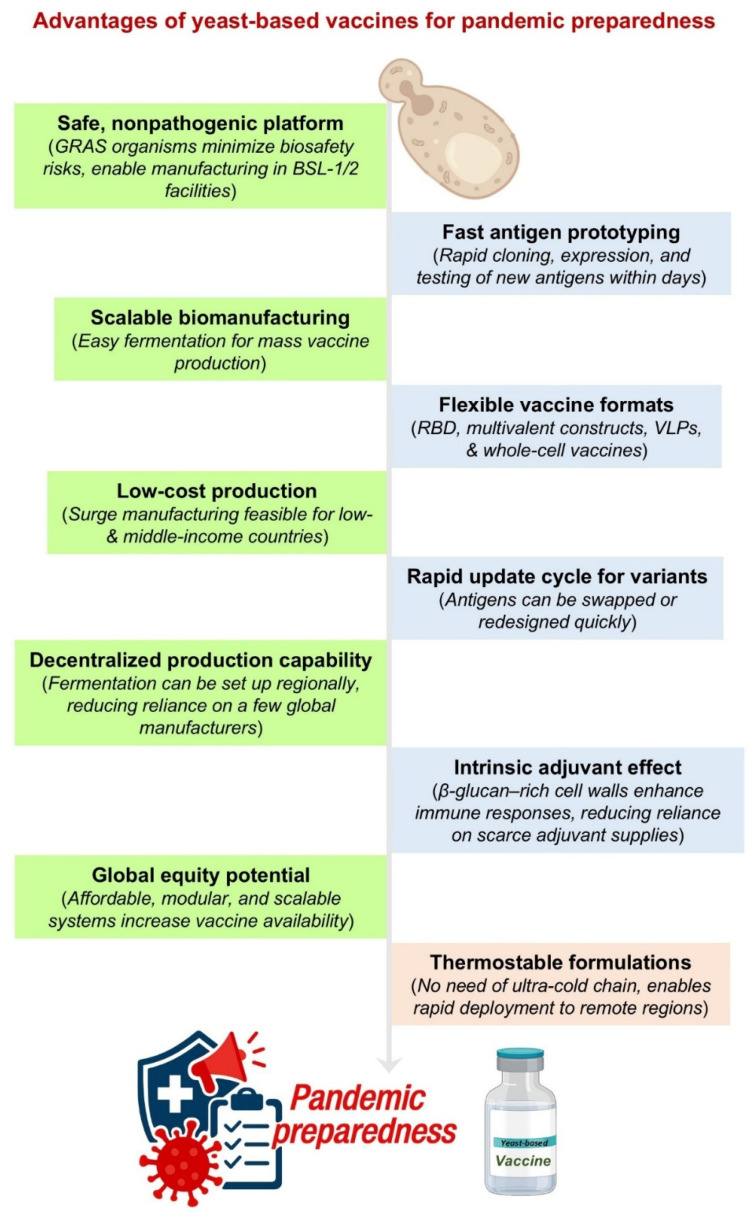
Key advantages of yeast-based vaccines for pandemic preparedness. This figure summarizes the practical and biological strengths that make yeast an attractive platform for rapid vaccine development. Yeast offers a safe, nonpathogenic system that supports scalable and low-cost biomanufacturing, decentralized production, and a fast transition from antigen design to testing. Its flexibility enables multiple vaccine formats, rapid variant updates, and intrinsic adjuvant activity. Thermostability and global affordability further position yeast-based vaccines as strong candidates for future pandemic response.

If the goal is breadth that survives viral drift, two ideas matter most, presenting conserved targets and keeping the platform modular so you can swap antigens without rebuilding the whole process. Interestingly, yeast helps on both counts (Figure 3). A practical example is the “plug-and-display” approach, where an RBD made in *P. pastoris* is clicked onto pre-made HBsAg nanoparticles using SpyTag/SpyCatcher chemistry. The HBsAg particles are already manufactured at GMP scale, and the RBD insert can be updated quickly. Notably, in animal studies, this RBD-on-HBsAg design was immunogenic and is inherently adaptable to variant or mosaic mixes [74].

Breadth can also come from multivalent or mosaic displays that push the immune system toward cross-reactive sites. Caltech’s mosaic RBD nanoparticles, which display RBDs from different sarbecoviruses on one particle, induced cross-clade neutralization and protection in animals. The concept is transferable to yeast pipelines because RBDs from multiple strains can be expressed in *Pichia* and attached to a common VLP chassis [75]. A second route is to target conserved regions outside the variable RBD tip. Prefusion-stabilized S2-only immunogens have now shown broad neutralization across sarbecoviruses and protection in mice, highlighting S2 as a realistic component for pan-coronavirus strategies. Interestingly, yeast hosts can express S2 subdomains and, with glycoengineering, present them with more human-like glycans [76]. Additionally, antigen engineering inside yeast is advancing in parallel. Yeast-produced RBD dimers with controlled glycans have improved potency and protection in challenge models, and the same workflow can update sequences as new variants appear. This is a simple way to increase valency and stabilize key epitopes without giving up the cost and scale benefits of fermentation [26]. Finally, yeast is also proving its value on the design side. Yeast-surface display and deep mutational scanning have mapped how spike and RBD mutations affect ACE2 binding and antibody escape, with newer datasets now covering XBB-class variants. These insights help identify antigen combinations that are harder for the virus to evade and inform the design of multivalent yeast-based VLPs [77]. Because the same yeast system can quickly manufacture updated RBD or S2 immunogens and decorate scalable VLP “chassis,” it offers a practical path toward variant-resilient or even pan-sarbecovirus vaccines that remain affordable and adaptable for regional production.

### 10.2. Application to Other Emerging Infectious Diseases

Yeast’s value extends well beyond COVID. Across influenza, RSV, and arboviruses, the platform has repeatedly shown that it can deliver complex antigens at scale, with the stability and cost profile needed for real-world deployment (Figure 3). *Pichia*-expressed influenza hemagglutinin subunits, including glycoengineered H7 and earlier H5 candidates, produced well-folded proteins that protected animals in challenge studies [78]. The platform has also handled hard targets like RSV F, where a prefusion-focused yeast antigen protected mice without signs of enhanced respiratory disease [79]. Next, for arboviruses, yeast can secrete Zika EDIII, produce dengue particles and EDIII constructs, and generate chikungunya VLPs at encouraging yields, an advantage for cost and surge manufacturing [80]. Overall, these case studies show that yeast is not pathogen-limited, rather, it is a versatile system that can support influenza, RSV, dengue, Zika, and chikungunya vaccines using stainless-steel fermentation, which many regions already operate. Conclusively, the combination of biological fit and accessible manufacturing makes yeast a practical, scalable option for the next wave of respiratory and mosquito-borne threats.

### 10.3. Yeast-Based Rapid Response Platforms for the Next Pandemic

Yeast offers a practical, modular, rapid-response platform for the next pandemic by combining fast antigen design with manufacturing systems that can turn on quickly across regions (Figure 3). Plug-and-display approaches let teams express an RBD or other antigen in *P. pastoris* and attach it to a ready-made HBsAg nanoparticle chassis, a pairing already produced under GMP and shown to be stable and immunogenic, exactly what is needed when speed matters [81,82]. Yeast vaccines are also compatible with 2–8 °C storage and the WHO CTC, easing last-mile delivery and fitting the realities of low-resource settings. In conclusion, the path is straightforward, invest now in pre-qualified yeast strains, vectors, and modular display systems so that, when the next threat emerges, programs can drop in a new antigen, run familiar fermentation, meet practical storage needs, and manufacture doses where they are needed most.

### 10.4. A Call for Greater Investment in Yeast-Based Vaccine Pipelines for Pandemic Preparedness

A stronger global vaccine strategy needs real investment in yeast-based platforms. COVID-19 already showed what they can deliver; a *Pichia*-expressed RBD vaccine reached the WHO Emergency Use Listing, and countries like Indonesia demonstrated that local yeast manufacturing is both feasible and effective [83]. What is missing now is commitment, not capability, notably, open, license-free toolkits like OPENPichia, paired with mature glycoengineering systems that already let regional manufacturers move faster and avoid the delays of MTAs (Material Transfer Agreements) and proprietary hosts [45]. Next, A GMP-validated VLP chassis, already produced at scale and shown to be stable and immunogenic, offers a plug-and-play path for variant or pan-sarbecovirus updates without rebuilding production lines. Additionally, as yeast vaccines can tolerate warmer temperatures under the WHO’s CTC, investment in label claims, thermostability analytics, and regional clinical networks made them practical for settings where cold chains are fragile. Further, techno-economic analyses consistently show that microbial recombinant platforms reach lower costs and need less capital than many cell-culture systems, making them ideal for large, affordable second-wave supply (Table 2 and Figure 3). Overall, yeast is not a fallback, but it is a scalable, modular, and globally accessible pillar that complements the speed of mRNA-based vaccines. The science is ready, the infrastructure exists, and focused investment now can turn scattered successes into a durable, equitable capability for the next pandemic.

## Figures and Tables

**Table 1:** Overview of yeast-based vaccine platforms for pandemic preparedness.

Purified Protein	VLPs	YSD	WRY
**Pros**			
No need for growth of the pathogenic entity	No need for the growth of a pathogenic entity	No need for protein purification	No need for protein purification
Safer compared to live or attenuated vaccine	Safer compared to live or attenuated vaccine	Rapid and fast manufacturing	Rapid and fast manufacturing
	Behave just like an attenuated virus	Cost effective	Cost effective
		No need for the growth of a pathogenic entity	No need for growth of the pathogenic entity
		Safer compared to live or attenuated vaccines	Safer compared to live or attenuated vaccine
		Potential for oral application	Potential for oral application
		No need of separate adjuvant	No need of separate adjuvant
**Cons**			
Purification of immunogenic protein can be costly and time-consuming	Purification of VLPs can be costly and time-consuming	People allergic to yeast are concerned	People allergic to yeast are concerned
Fast body clearance	Require proper and controlled storage conditions		
Need adjuvant			
Require proper and controlled storage conditions			
Poor immunogenicity			

**Table 2:** Comparison of major vaccine platforms.

	Yeast	mRNA	Adenovirus	Animal Cell Line	Plants
**Production cost**	Low	High	High	High	Moderate
**Growth media**	Simple		Complex	Complex	Simple
**Scale up**	Easy		Difficult	Difficult	Difficult
**Storage**	Can be stored at room temperature	Need a low to deep freezer	Need a low to deep freezer	Need a low to deep freezer	Can be stored at room temperature
**Ways the platform can be used**	Multiple	Only one way	Only one way	Only one way	Only one way
**Oral application**	Can be done	no	no	no	Can be done
**Need of adjuvant**	Maybe needed	Must	Maybe needed	Must	Must
**Status**	In public use	In public use	In public use	In public use	No use in public so far
**Nature of immunogen**	Protein	mRNA (nucleic acid)	Protein	Protein	Protein
**Immunogenicity**	High	Low	High	High	High
**Post translational modification of immunogen**	Possible	No	Possible	Possible	Possible

**Table 3:** Selected yeast-based vaccines, approved or under clinical trials.

Vaccine	Yeast Species	Platform	Disease/Pathogen	Regulatory Status/Clinical Trial
**Corbevax**	*P. pastoris*	Protein subunit	SARS-CoV-2	Approved
**Abdala**	*S. cerevisiae*	Protein subunit	SARS-CoV-2	Approved
**Gardasil9^®^**	*S. cerevisiae*	VLPs	HPV	Approved
**Mosquirix™**	*S. cerevisiae*	VLPs	Malaria	Approved
**NA**	*S. cerevisiae*	VLPs	HIV	Phase-1 (NCT00001053)
**Hepavax-Gene**	*H. polymorpha*	VLPs	Hepatitis B	Licensed
**Fendrix**	*S. cerevisiae*	VLPs	Hepatitis B	Licensed
**Heplisav-B**	*H. polymorpha*	VLPs	Hepatitis B	Licensed
**Engerix**	*S. cerevisiae*	VLPs	Hepatitis B	Licensed
**Recombivax HB ** **(H-B-Vac^®^-II)**	*S. cerevisiae*	VLPs	Hepatitis B	Licensed
**Brachyury (GI-6301)**	*S. cerevisiae*	Protein subunit	Cancer	Phase-II (NCT02383498)

## Data Availability

There is no data associated with this manuscript.

## References

[1] Anderer S. (2025). COVID-19 Vaccines Averted 2.5 Million Deaths, Mostly Among Older Adults. JAMA.

[2] Ioannidis J.P.A., Pezzullo A.M., Cristiano A., Boccia S. (2025). Global Estimates of Lives and Life-Years Saved by COVID-19 Vaccination During 2020–2024. JAMA Health Forum.

[3] Conservation and Enhanced Binding of SARS-CoV-2 Omicron Spike Protein to Coreceptor Neuropilin-1 Predicted by Docking Analysis—PubMed. https://pubmed.ncbi.nlm.nih.gov/35447881/.

[4] Kumar R., Kumar P. (2019). Yeast-Based Vaccines: New Perspective in Vaccine Development and Application. FEMS Yeast Res..

[5] Kumar R., Srivastava V., Ahmad A., Mandal S.M., Baindara P. (2025). Whole Recombinant Yeast-Based Vaccines: Concept, Importance, Issues, and Future Scope. Crit. Rev. Biotechnol..

[6] Zang J., Zhu Y., Zhou Y., Gu C., Yi Y., Wang S., Xu S., Hu G., Du S., Yin Y. (2021). Yeast-Produced RBD-Based Recombinant Protein Vaccines Elicit Broadly Neutralizing Antibodies and Durable Protective Immunity against SARS-CoV-2 Infection. Cell Discov..

[7] Kumar R. (2025). Recombinant Yeast-Based Vaccines: Importance and Applications. Infect. Dis. Rep..

[8] Lang Q., Huang N., Li L., Liu K., Chen H., Liu X., Ge L., Yang X. (2025). Novel and Efficient Yeast-Based Strategies for Subunit Vaccine Delivery against COVID-19. Int. J. Biol. Macromol..

[9] Pollet J., Chen W.-H., Versteeg L., Keegan B., Zhan B., Wei J., Liu Z., Lee J., Kundu R., Adhikari R. (2021). SARS-CoV-2 RBD219-N1C1: A Yeast-Expressed SARS-CoV-2 Recombinant Receptor-Binding Domain Candidate Vaccine Stimulates Virus Neutralizing Antibodies and T-Cell Immunity in Mice. Hum. Vaccines Immunother..

[10] Si C., Bai J., Li Y., Li Y., Liu Y., Zhou X., Shi J., Nakanishi H., Li Z. (2025). Establishment of a Novel Platform for Developing Oral Vaccines Based on the Surface Display System of Yeast Spores. Int. J. Mol. Sci..

[11] Maneira C., Chamas A., Lackner G. (2025). Engineering *Saccharomyces cerevisiae* for Medical Applications. Microb. Cell Factories.

[12] Danzelle C., Cunha P., Noleto P.G., Gilbert F.B., Santos K.R., Staub C., Pinard A., Deslis A., Barbey S., Germon P. (2024). *Saccharomyces cerevisiae* as a Platform for Vaccination against Bovine Mastitis. Vaccine.

[13] Li H., Hua D., Qu Q., Cao H., Feng Z., Liu N., Huang J., Zhang L. (2023). Oral Immunization with Recombinant *Saccharomyces cerevisiae* Expressing Viral Capsid Protein 2 of Infectious Bursal Disease Virus Induces Unique Specific Antibodies and Protective Immunity. Vaccines.

[14] de Sá Magalhães S., Keshavarz-Moore E. (2021). *Pichia pastoris* (*Komagataella phaffii*) as a Cost-Effective Tool for Vaccine Production for Low- and Middle-Income Countries (LMICs). Bioengineering.

[15] Karbalaei M., Rezaee S.A., Farsiani H. (2020). *Pichia pastoris*: A Highly Successful Expression System for Optimal Synthesis of Heterologous Proteins. J. Cell. Physiol..

[16] Kuruti K., Vittaladevaram V., Urity S.V., Palaniappan P., Bhaskar R.U. (2020). Evolution of *Pichia pastoris* as a Model Organism for Vaccines Production in Healthcare Industry. Gene Rep..

[17] Ergün B.G., Berrios J., Binay B., Fickers P. (2021). Recombinant Protein Production in *Pichia pastoris*: From Transcriptionally Redesigned Strains to Bioprocess Optimization and Metabolic Modelling. FEMS Yeast Res..

[18] Laukens B., De Wachter C., Callewaert N. (2015). Engineering the *Pichia pastoris* N-Glycosylation Pathway Using the GlycoSwitch Technology. Glyco-Engineering. Methods in Molecular Biology.

[19] McAleer W.J., Buynak E.B., Maigetter R.Z., Wampler D.E., Miller W.J., Hilleman M.R. (1984). Human Hepatitis B Vaccine from Recombinant Yeast. Nature.

[20] Pattyn J., Hendrickx G., Vorsters A., Van Damme P. (2021). Hepatitis B Vaccines. J. Infect. Dis..

[21] Full Article: Hepatitis B Vaccine Development and Implementation. https://www.tandfonline.com/doi/full/10.1080/21645515.2020.1732166?utm_source=chatgpt.com.

[22] Stubbs A.C., Martin K.S., Coeshott C., Skaates S.V., Kuritzkes D.R., Bellgrau D., Franzusoff A., Duke R.C., Wilson C.C. (2001). Whole Recombinant Yeast Vaccine Activates Dendritic Cells and Elicits Protective Cell-Mediated Immunity. Nat. Med..

[23] Ardiani A., Higgins J.P., Hodge J.W. (2010). Vaccines Based on Whole Recombinant *Saccharomyces cerevisiae* Cells. FEMS Yeast Res..

[24] Limonta-Fernández M., Chinea-Santiago G., Martín-Dunn A.M., Gonzalez-Roche D., Bequet-Romero M., Marquez-Perera G., González-Moya I., Canaan-Haden-Ayala C., Cabrales-Rico A., Espinosa-Rodríguez L.A. (2022). An Engineered SARS-CoV-2 Receptor-Binding Domain Produced in *Pichia pastoris* as a Candidate Vaccine Antigen. New Biotechnol..

[25] Liu Y., Zhao D., Wang Y., Chen Z., Yang L., Li W., Gong Y., Gan C., Tang J., Zhang T. (2022). A Vaccine Based on the Yeast-Expressed Receptor-Binding Domain (RBD) Elicits Broad Immune Responses against SARS-CoV-2 Variants. Front. Immunol..

[26] Zhao T., Liu S., Wang P., Zhang Y., Kang X., Pan X., Li L., Li D., Gao P., An Y. (2024). Protective RBD-Dimer Vaccines against SARS-CoV-2 and Its Variants Produced in Glycoengineered *Pichia pastoris*. PLoS Pathog..

[27] Gao T., Ren Y., Li S., Lu X., Lei H. (2021). Immune Response Induced by Oral Administration with a *Saccharomyces cerevisiae*-Based SARS-CoV-2 Vaccine in Mice. Microb. Cell Factories.

[28] Yeast-Based Virus-like Particles as an Emerging Platform for Vaccine Development and Delivery—PubMed. https://pubmed.ncbi.nlm.nih.gov/36851356/.

[29] Zhao Q., Li S., Yu H., Xia N., Modis Y. (2013). Virus-like Particle-Based Human Vaccines: Quality Assessment Based on Structural and Functional Properties. Trends Biotechnol..

[30] Hao X., Yuan F., Yao X. (2024). Advances in Virus-like Particle-Based SARS-CoV-2 Vaccines. Front. Cell. Infect. Microbiol..

[31] Kingston N.J., Snowden J.S., Martyna A., Shegdar M., Grehan K., Tedcastle A., Pegg E., Fox H., Macadam A.J., Martin J. (2023). Production of Antigenically Stable Enterovirus A71 Virus-like Particles in *Pichia pastoris* as a Vaccine Candidate. BioRxiv.

[32] Xing H., Zhu L., Wang P., Zhao G., Zhou Z., Yang Y., Zou H., Yan X. (2022). Display of Receptor-Binding Domain of SARS-CoV-2 Spike Protein Variants on the *Saccharomyces cerevisiae* Cell Surface. Front. Immunol..

[33] Lopez-Morales J., Vanella R., Utzinger T., Schittny V., Hirsiger J., Osthoff M., Berger C.T., Guri Y., Nash M.A. (2023). Multiplexed On-Yeast Serological Assay for Immune Escape Screening of SARS-CoV-2 Variants. iScience.

[34] Development of a Yeast Cell Surface Display Method Using the SpyTag/SpyCatcher System|Scientific Reports. https://www.nature.com/articles/s41598-021-90593-w.

[35] de Macêdo L.S., Espinoza B.C.F., Invenção M.d.C.V., de Pinho S.S., Leal L.R.S., Silva M.E.D.S., Bandeira B.M.A., Novis P.V.S., Souza T.H.D.S., Maux J.M.d.L. (2025). Oral Immunization with Yeast-Surface Display of SARS-CoV-2 Antigens in *Pichia pastoris* Induces Humoral Responses in BALB/C Mice. Infect. Dis. Rep..

[36] Park H.-Y., Yoon H.-K., Kim J.-Y., Park S.-R. (2018). Heat-Killed *Saccharomyces cerevisiae*, A Dectin-1 Agonist, Selectively Induces IgG4 Production by Human B Cells. Immune Netw..

[37] Cohn A., Morse M.A., O’Neil B., Whiting S., Coeshott C., Ferraro J., Bellgrau D., Apelian D., Rodell T.C. (2018). Whole Recombinant *Saccharomyces cerevisiae* Yeast Expressing Ras Mutations as Treatment for Patients with Solid Tumors Bearing Ras Mutations: Results From a Phase 1 Trial. J. Immunother..

[38] Oligonucleotide-Based CRISPR-Cas9 Toolbox for Efficient Engineering of *Komagataella phaffii*|FEMS Yeast Research|Oxford Academic. https://academic.oup.com/femsyr/article/doi/10.1093/femsyr/foae026/7740463.

[39] Zhou W., Li Y., Liu G., Qin W., Wei D., Wang F., Gao B. (2024). CRISPR/Cas9-Based Toolkit for Rapid Marker Recycling and Combinatorial Libraries in *Komagataella phaffii*. Appl. Microbiol. Biotechnol..

[40] Bai F., Cai P., Yao L., Shen Y., Li Y., Zhou Y.J. (2025). Inducible Regulating Homologous Recombination Enables Precise Genome Editing in *Pichia pastoris* without Perturbing Cellular Fitness. Trends Biotechnol..

[41] Wu L.-Y., Xu Y., Yu X.-W. (2024). Efficient CRISPR-Mediated C-to-T Base Editing in *Komagataella phaffii*. Biotechnol. J..

[42] Weber Y., Böck D., Ivașcu A., Mathis N., Rothgangl T., Ioannidi E.I., Blaudt A.C., Tidecks L., Vadovics M., Muramatsu H. (2024). Enhancing Prime Editor Activity by Directed Protein Evolution in Yeast. Nat. Commun..

[43] Feng Q., Ning X., Qin L., Li J., Li C. (2023). Quantitative and Modularized CRISPR/dCas9-dCpf1 Dual Function System in *Saccharomyces cerevisiae*. Front. Bioeng. Biotechnol..

[44] Tafrishi A., Trivedi V., Xing Z., Li M., Mewalal R., Cutler S.R., Blaby I., Wheeldon I. (2024). Functional Genomic Screening in *Komagataella phaffii* Enabled by High-Activity CRISPR-Cas9 Library. Metab. Eng..

[45] Claes K., Van Herpe D., Vanluchene R., Roels C., Van Moer B., Wyseure E., Vandewalle K., Eeckhaut H., Yilmaz S., Vanmarcke S. (2024). OPENPichia: Licence-Free *Komagataella phaffii* Chassis Strains and Toolkit for Protein Expression. Nat. Microbiol..

[46] Smirnov K., Weiss F., Hatzl A.-M., Rieder L., Olesen K., Jensen S., Glieder A. (2024). Comparison of CRISPR-MAD7 and CRISPR-Cas9 for Gene Disruptions in *Komagataella phaffii*. J. Fungi.

[47] Singh R.P., Bhardwaj A. (2023). β-Glucans: A Potential Source for Maintaining Gut Microbiota and the Immune System. Front. Nutr..

[48] Panão-Costa J., Colaço M., Jesus S., Lebre F., Cruz M.T., Alfaro-Moreno E., Borges O. (2025). Yeast-Derived Glucan Particles: Biocompatibility, Efficacy, and Immunomodulatory Potential as Adjuvants and Delivery Systems. Pharmaceutics.

[49] Azevedo-Silva J., Amorim M., Tavares-Valente D., Sousa P., Mohamath R., Voigt E.A., Guderian J.A., Kinsey R., Viana S., Reis F. (2024). Exploring Yeast Glucans for Vaccine Enhancement: Sustainable Strategies for Overcoming Adjuvant Challenges in a SARS-CoV-2 Model. Eur. J. Pharm. Biopharm..

[50] Kumar R., Kharbikar B.N. (2021). Lyophilized Yeast Powder for Adjuvant Free Thermostable Vaccine Delivery. Appl. Microbiol. Biotechnol..

[51] Bachmann M.F., van Damme P., Lienert F., Schwarz T.F. (2025). Virus-like Particles: A Versatile and Effective Vaccine Platform. Expert Rev. Vaccines.

[52] Wouters O.J., Shadlen K.C., Salcher-Konrad M., Pollard A.J., Larson H.J., Teerawattananon Y., Jit M. (2021). Challenges in Ensuring Global Access to COVID-19 Vaccines: Production, Affordability, Allocation, and Deployment. Lancet.

[53] Viral Vectored Vaccines: Design, Development, Preventive and Therapeutic Applications in Human Diseases|Signal Transduction and Targeted Therapy. https://www.nature.com/articles/s41392-023-01408-5.

[54] Sanchez-Martinez Z.V., Alpuche-Lazcano S.P., Stuible M., Durocher Y. (2024). CHO Cells for Virus-like Particle and Subunit Vaccine Manufacturing. Vaccine.

[55] Hong Q., Liu J., Wei Y., Wei X. (2023). Application of Baculovirus Expression Vector System (BEVS) in Vaccine Development. Vaccines.

[56] Corbett K.S., Edwards D.K., Leist S.R., Abiona O.M., Boyoglu-Barnum S., Gillespie R.A., Himansu S., Schäfer A., Ziwawo C.T., DiPiazza A.T. (2020). SARS-CoV-2 mRNA Vaccine Design Enabled by Prototype Pathogen Preparedness. Nature.

[57] Meyer S. Update on CDC’s COVID-19 Vaccine Safety Monitoring. https://www.cdc.gov/acip/downloads/slides-2025-06-25-26/04-Meyer-COVID-508.pdf.

[58] Davidopoulou C., Kouvelas D., Ouranidis A. (2024). COMPARING Vaccine Manufacturing Technologies Recombinant DNA vs in Vitro Transcribed (IVT) mRNA. Sci. Rep..

[59] Oude Blenke E., Örnskov E., Schöneich C., Nilsson G.A., Volkin D.B., Mastrobattista E., Almarsson Ö., Crommelin D.J.A. (2023). The Storage and In-Use Stability of mRNA Vaccines and Therapeutics: Not a Cold Case. J. Pharm. Sci..

[60] Parums D.V. (2022). Editorial: First Approval of the Protein-Based Adjuvanted Nuvaxovid (NVX-CoV2373) Novavax Vaccine for SARS-CoV-2 Could Increase Vaccine Uptake and Provide Immune Protection from Viral Variants. Med. Sci. Monit. Int. Med. J. Exp. Clin. Res..

[61] Flublok 2024–2025 (Influenza Vaccine Injection for Intramuscular Use): Side Effects, Uses, Dosage, Interactions, Warnings. https://www.rxlist.com/flublok-2024-2025-drug.htm.

[62] Block J.P., Boehmer T.K., Forrest C.B., Carton T.W., Lee G.M., Ajani U.A., Christakis D.A., Cowell L.G., Draper C., Ghildayal N. (2022). Cardiac Complications After SARS-CoV-2 Infection and mRNA COVID-19 Vaccination—PCORnet, United States, January 2021–January 2022. MMWR Morb. Mortal. Wkly. Rep..

[63] Thrombotic Thrombocytopenia after ChAdOx1 nCov-19 Vaccination|New England Journal of Medicine. https://www.nejm.org/doi/full/10.1056/NEJMoa2104840.

[64] Fix J., Christopher Mast T., Smith K., Baker N. (2024). Benefit-Risk Assessment for the Novavax COVID-19 Vaccine (NVX-CoV2373). Vaccine.

[65] Cid R., Bolívar J. (2021). Platforms for Production of Protein-Based Vaccines: From Classical to Next-Generation Strategies. Biomolecules.

[66] Pan Y., Yang J., Wu J., Yang L., Fang H. (2022). Current Advances of *Pichia pastoris* as Cell Factories for Production of Recombinant Proteins. Front. Microbiol..

[67] Buckland B., Sanyal G., Ranheim T., Pollard D., Searles J.A., Behrens S., Pluschkell S., Josefsberg J., Roberts C.J. (2024). Vaccine Process Technology—A Decade of Progress. Biotechnol. Bioeng..

[68] Hotez P.J., Bottazzi M.E. (2020). Developing a Low-Cost and Accessible COVID-19 Vaccine for Global Health. PLoS Negl. Trop. Dis..

[69] COVID-19 Vaccines with WHO Emergency Use Listing|WHO—Prequalification of Medical Products (IVDs, Medicines, Vaccines and Immunization Devices, Vector Control). https://extranet.who.int/prequal/vaccines/covid-19-vaccines-who-emergency-use-listing.

[70] Hernández-Bernal F., Ricardo-Cobas M.C., Martín-Bauta Y., Rodríguez-Martínez E., Urrutia-Pérez K., Urrutia-Pérez K., Quintana-Guerra J., Navarro-Rodríguez Z., Piñera-Martínez M., Rodríguez-Reinoso J.L. (2023). A Phase 3, Randomised, Double-Blind, Placebo-Controlled Clinical Trial Evaluation of the Efficacy and Safety of a SARS-CoV-2 Recombinant Spike RBD Protein Vaccine in Adults (ABDALA-3 Study). Lancet Reg. Health-Am..

[71] Lemos-Pérez G., Barrese-Pérez Y., Chacón-Quintero Y., Uranga-Piña R., Avila-Albuerne Y., Figueroa-García I., Calderín-Marín O., Gómez-Vázquez M.M., Piñera-Martínez M., Chávez-Valdés S. (2024). Safety and Immunogenicity of the Intranasal Vaccine Candidate Mambisa and the Intramuscular Vaccine Abdala Used as Booster Doses for COVID-19 Convalescents: A Randomized Phase 1–2 Clinical Trial. Vaccines.

[72] Chen W.-H., Pollet J., Strych U., Lee J., Liu Z., Kundu R.T., Versteeg L., Villar M.J., Adhikari R., Wei J. (2022). Yeast-Expressed Recombinant SARS-CoV-2 Receptor Binding Domain RBD203-N1 as a COVID-19 Protein Vaccine Candidate. Protein Expr. Purif..

[73] Liu J., Wang T., Ren H., Liu R., Wang Q., Wu J., Liu B. (2025). XBB.1.5 RBD-Based Bivalent Vaccines Induced Antibody Responses Against SARS-CoV-2 Variants in Mice. Vaccines.

[74] SARS-CoV-2 Receptor Binding Domain Displayed on HBsAg Virus–like Particles Elicits Protective Immunity in Macaques|Science Advances. https://www.science.org/doi/10.1126/sciadv.abl6015.

[75] Mosaic RBD Nanoparticles Protect against Challenge by Diverse Sarbecoviruses in Animal Models|Science. https://www.science.org/doi/10.1126/science.abq0839.

[76] Hsieh C.-L., Leist S.R., Miller E.H., Zhou L., Powers J.M., Tse A.L., Wang A., West A., Zweigart M.R., Schisler J.C. (2024). Prefusion-Stabilized SARS-CoV-2 S2-Only Antigen Provides Protection against SARS-CoV-2 Challenge. Nat. Commun..

[77] Starr T.N., Greaney A.J., Hilton S.K., Ellis D., Crawford K.H.D., Dingens A.S., Navarro M.J., Bowen J.E., Tortorici M.A., Walls A.C. (2020). Deep Mutational Scanning of SARS-CoV-2 Receptor Binding Domain Reveals Constraints on Folding and ACE2 Binding. Cell.

[78] Athmaram T.N., Saraswat S., Santhosh S.R., Singh A.K., Suryanarayana W.S., Priya R., Gopalan N., Parida M., Rao P.V.L., Vijayaraghavan R. (2011). Yeast Expressed Recombinant Hemagglutinin Protein of Novel H1N1 Elicits Neutralising Antibodies in Rabbits and Mice. Virol. J..

[79] Li H., Cao L., Zhang Y., Ren H., Zhao P., Xu W. (2020). Human Respiratory Syncytial Virus F Protein Expressed in *Pichia pastoris* or Escherichia Coli Induces Protective Immunity without Inducing Enhanced Respiratory Disease in Mice. Arch. Virol..

[80] Zhang W., Qu P., Li D., Zhang C., Liu Q., Zou G., Dupont-Rouzeyrol M., Lavillette D., Jin X., Yin F. (2019). Yeast-Produced Subunit Protein Vaccine Elicits Broadly Neutralizing Antibodies That Protect Mice against Zika Virus Lethal Infection. Antivir. Res..

[81] Marini A., Zhou Y., Li Y., Taylor I.J., Leneghan D.B., Jin J., Zaric M., Mekhaiel D., Long C.A., Miura K. (2019). A Universal Plug-and-Display Vaccine Carrier Based on HBsAg VLP to Maximize Effective Antibody Response. Front. Immunol..

[82] Dalvie N.C., Tostanoski L.H., Rodriguez-Aponte S.A., Kaur K., Bajoria S., Kumru O.S., Martinot A.J., Chandrashekar A., McMahan K., Mercado N.B. (2021). A Modular Protein Subunit Vaccine Candidate Produced in Yeast Confers Protection against SARS-CoV-2 in Non-Human Primates. BioRxiv.

[83] Nurdin A., Movieta Nency Y., Maddeppungeng M., Sekartini R., Mulia Sari R., Surachman F., Fitry Yani F., Raveinal, Anggrainy F., Hafiz A. (2024). Immunogenicity and Safety of SARS-CoV-2 Recombinant Protein Subunit Vaccine (IndoVac) Adjuvanted with Alum and CpG 1018 in Indonesian Adults: A Phase 3, Randomized, Active-Controlled, Multicenter Trial. Vaccine.

